# Uncovering the Role of Hydroxycinnamoyl Transferase in Boosting Chlorogenic Acid Accumulation in *Carthamus tinctorius* Cells under Methyl Jasmonate Elicitation

**DOI:** 10.3390/ijms25052710

**Published:** 2024-02-27

**Authors:** Zebo Liu, Xiaofeng Zhu, Ali Mohsin, Huijie Sun, Linxiao Du, Zhongping Yin, Yingping Zhuang, Meijin Guo

**Affiliations:** 1 State Key Laboratory of Bioreactor Engineering, East China University of Science and Technology, Shanghai 200237, China; 2 Jiangxi Key Laboratory of Natural Products and Functional Foods, Jiangxi Agricultural University, Nanchang 330045, China

**Keywords:** chlorogenic acids biosynthetic regulation, *Carthamus tinctorius* suspension cells, hydroxycinnamoyl transferase, functional characterization, methyl jasmonate

## Abstract

Chlorogenic acids (CGAs) are bioactive compounds widely used in the food, pharmaceutical, and cosmetic industries. *Carthamus tinctorius* is an important economic crop, and its suspension cells are rich in CGAs. However, little is known about the biosynthesis and regulation of CGAs in *Carthamus tinctorius* cells. This study first elucidated the regulatory mechanism of CGA biosynthesis in methyl jasmonate (MeJA)-treated *Carthamus tinctorius* cells and the role of the MeJA-responsive *hydroxycinnamoyl transferase* (*HCT*) gene in enhancing their CGA accumulation. Firstly, temporal changes in intracellular metabolites showed that MeJA increased the intracellular CGA content up to 1.61-fold to 100.23 mg·g^−1^. Meanwhile, 31 primary metabolites showed significant differences, with 6 precursors related to increasing CGA biosynthesis. Secondly, the transcriptome data revealed 3637 new genes previously unannotated in the *Carthamus tinctorius* genome and 3653 differentially expressed genes. The genes involved in the plant signaling pathway and the biosynthesis of CGAs and their precursors showed a general up-regulation, especially the *HCT* gene family, which ultimately promoted CGA biosynthesis. Thirdly, the expression of a newly annotated and MeJA-responsive *HCT* gene (*CtHCT*, CtNewGene_3476) was demonstrated to be positively correlated with CGA accumulation in the cells, and transient overexpression of *CtHCT* enhanced CGA accumulation in tobacco. Finally, in vitro catalysis kinetics and molecular docking simulations revealed the ability and mechanism of the *Ct*HCT protein to bind to various substrates and catalyze the formation of four hydroxycinnamic esters, including CGAs. These findings strengthened our understanding of the regulatory mechanism of CGA biosynthesis, thereby providing theoretical support for the efficient production of CGAs.

## 1. Introduction

Chlorogenic acids (CGAs) are a class of hydroxycinnamates that are unique to plants. They are represented by caffeoyl quinic acids (mono- and multi-caffeoyl quinic acids) [1]. These compounds have been shown to possess various bioactive functions. They have been found to inhibit the proliferation of cancer cells, bacteria, and viruses. Additionally, they have hepatoprotective and neuroprotective effects, as well as significant antioxidant and UV-protective capabilities [1,2,3]. As a result, CGAs are valuable functional factors with broad applications in the food, pharmaceutical, and cosmetic industries [4,5,6].

*Carthamus tinctorius* (*C. tinctorius*), also known as safflower, is a promising and versatile industrial crop used in pharmaceuticals, dyestuffs, edible oil products, and animal feeds [7]. In our previous study, a cell suspension culture system was developed for *C. tinctorius* to obtain this plant resource more efficiently [8]. Interestingly, the suspension cells of *C. tinctorius* were found to contain high levels of CGAs, mainly composed of mono-, di-, and tri-caffeoyl quinic acids. Upon elicitation with methyl jasmonate (MeJA), the CGA content in the cells reached up to 10% of the cell dry weight [8], which was higher than the CGA content reported in other plant cells such as *Lonicera japonica* and *Bidens pilosa* [9,10]. Therefore, *C. tinctorius* cells are a promising new material to study the biosynthetic regulatory mechanisms of CGAs due to their ability to accumulate high levels of these compounds. Previous studies on the biosynthetic regulation mechanism of bioactive compounds in *C. tinctorius* have mainly focused on flavonoids, anthocyanins, and polyunsaturated fatty acids [11,12,13]. However, the biosynthetic regulatory mechanisms of CGAs in *C. tinctorius* remain unknown. This hinders the efficient production of CGAs and underscores the importance of elucidating their biosynthetic regulation mechanism in the cells.

The biosynthesis pathway of CGAs has been investigated in various species, such as *Cecropia obtusifolia*, *Lonicera maackii*, *Ipomoea batatas*, and *Echinacea purpurea* [14,15,16,17]. However, due to the complexity of CGA biosynthesis, there is ongoing debate regarding their precise biosynthetic mechanisms [18]. Initially, phenylalanine ammonia-lyase (PAL) deaminates phenylalanine to form cinnamic acid. Subsequently, five known pathways exist for CGA biosynthesis (Figure S1) [1,2,3,19,20]. The first pathway involves the formation of *p*-coumaroyl CoA from cinnamic acid by cinnamic acid 4-hydroxylase (C4H) and 4-coumarate-CoA ligase (4CL), followed by the consecutive catalysis of CYP98A (C3’H) and hydroxycinnamoyl transferase/hydroxycinnamoyl-CoA, quinate transferase (HCT/HQT), to produce caffeoyl quinic acid (CQA). The second pathway generates caffeic acid from *p*-coumaroyl CoA by C3’H, HCT/HQT and caffeoylshikimate esterase (CSE), followed by its conversion into CQA by 4CL and HCT/HQT. The third pathway directly produces CQA from *p*-coumaroyl quinic acid by C3’H. The fourth pathway synthesizes caffeic acid from *p*-coumaric acid by *p*-coumarate 3-hydroxylase (C3H), followed by the action of 4CL and HCT/HQT to produce CQA. In contrast, the fifth pathway is characterized by the glycosylation of cinnamic acid by UGT84A20 (UGCT) to form cinnamoyl D-glucose, which is then converted into CQA by a specific acyltransferase. In all of the above pathways, acyltransferases, especially HCT/HQT, are necessary for generating hydroxycinnamic esters, which serve as precursors or end products in the formation of CGAs [19]. Therefore, acyltransferases play a vital and irreplaceable role in the biosynthesis of CGAs.

Hydroxycinnamoyl transferase (HCT) and hydroxycinnamoyl-CoA:quinate transferase (HQT) are important members of the plant BAHD acyltransferase superfamily [21]. HCT catalyzes the formation of hydroxycinnamic esters using various acyl acceptors (e.g., shikimic acid and quinic acid) and acyl donors (e.g., cinnamoyl-CoA and *p*-coumaroyl CoA) as substrates, while HQT prefers quinic acid as an acyl acceptor over shikimic acid [22,23]. HCT regulates the metabolic flux of *p*-coumaroyl CoA and thus mediates the biosynthesis of phenylpropanoid derivatives, including chlorogenic acids, monolignins, and flavonoids. For example, the overexpression of *Camellia sinensis*-derived *CsHCT1* and *CsHCT2* in *Arabidopsis thaliana* and *Nicotiana tabacum* significantly increased the accumulation of chlorogenic acids and monolignins but decreased the accumulation of flavonoids [24]. In some plants, CGA biosynthesis is mainly limited by HQT, which facilitates efficient acyl transfer between quinic acid and caffeoyl-CoA. For instance, manipulating *HQT* expression in *Solanum lycopersicum* leaves resulted in significant changes in the content of CGAs [25]. However, CGAs can still accumulate in some plants that lack HQT but possess HCT, such as *Camellia sinensis* and *Gardenia jasminoides* [24,26]. This suggests that HCT may compensate for HQT in CGA biosynthesis, thereby meeting the growth and developmental needs of plants through alternative pathways. Currently, the role of HCT in CGA biosynthesis in *C. tinctorius* is still unclear. Therefore, elucidating the function of HCT is crucial for a comprehensive understanding of the diversity and flexibility of CGA biosynthesis pathways in plants, especially in *C. tinctorius*.

This study aims to uncover the regulatory mechanism of CGA biosynthesis in *C. tinctorius* cells under MeJA elicitation and the role of MeJA-responsive *HCT* gene in promoting their CGA accumulation. Firstly, high-performance liquid chromatography and gas chromatography-mass spectrometry were used to analyze the temporal changes of CGAs and primary metabolites in the cells. Secondly, strand-specific transcriptome sequencing was performed to identify new genes that were not annotated in the *C. tinctorius* genome and to investigate the transcriptional response of the cells. Finally, various methods, including bioinformatics, transient expression, prokaryotic protein expression, in vitro catalysis kinetics, and molecular docking simulation, were used to analyze the role of the newly annotated and MeJA-responsive *HCT* gene in CGA accumulation in the cells. Our findings will provide theoretical support for the efficient production and further development of CGAs in *C. tinctorius* cells.

## 2. Results

### 2.1. Temporal Effect of Methyl Jasmonate on Intracellular Metabolites in C. tinctorius Cells

#### 2.1.1. Promoting Effect on Chlorogenic Acid Accumulation

**Figure 1:**
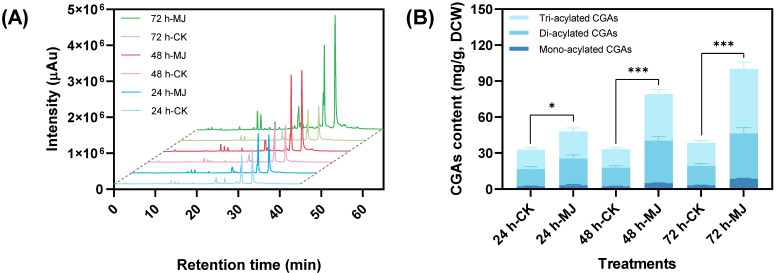
Temporal effects of methyl jasmonate (MeJA) on the accumulation of CGAs in *C. tinctorius* cells: (**A**) HPLC detection; (**B**) CGA content (* *p* < 0.05 and *** *p* < 0.001 indicate statistical significance levels from a *t*-test).

The chlorogenic acids (CGAs) in *C. tinctorius* cells have been classified into three types: mono-, di-, and tri-acylated CGAs [8]. As shown in Figure 1A, the addition of methyl jasmonate (MeJA, 100 µM) significantly increased the peak area of CGAs in the cell extracts. The total CGA content in the MeJA-treated group increased significantly with the extension of the co-culture time (24–72 h). The highest total CGA content (100.23 mg·g^−1^) was observed after 72 h of elicitation (Figure 1B), which was 2.61 times that of the control group. The temporal changes in the content of different types of CGAs were basically consistent with that of the total CGAs. Furthermore, the proportion of different types of CGAs in descending order was tri-acylated CGAs, di-acylated CGAs, and mono-acylated CGAs, with the percentage of tri-acylated CGAs ranging from 46.66% to 53.85% (Figure 1B). Therefore, MeJA elicitation significantly increased the accumulation of CGAs in *C. tinctorius* cells.

#### 2.1.2. Metabolic Profile Changes of Primary Metabolites

**Figure 2:**
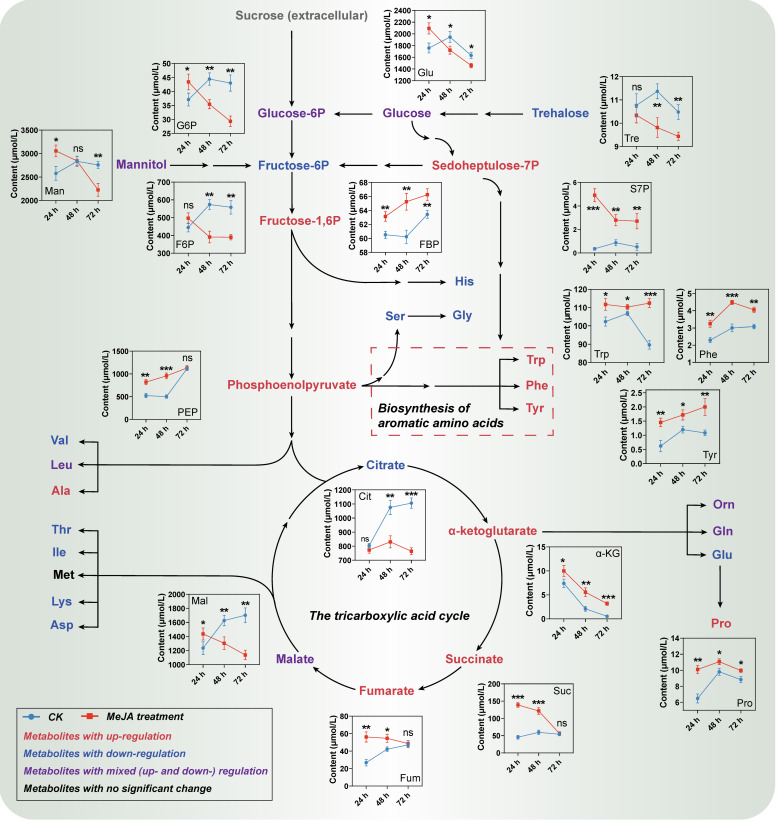
Temporal changes in metabolites of both central carbon metabolism and amino acid biosynthesis pathways in MeJA-treated *C. tinctorius* cells [the abbreviation “P” in the compound name represents the phosphate group; * *p* < 0.05, ** *p* < 0.01, *** *p* < 0.001, and ns (non-significant) indicate statistical significance levels from a *t*-test.

Primary metabolites, including carbohydrates, organic acids, and amino acids, serve as crucial precursors and energy sources for the biosynthesis of secondary metabolites [27]. This study revealed significant changes in the content of seven carbohydrates and six organic acids, which were mainly involved in the central carbon metabolic pathways, i.e., glycolysis, pentose phosphate, and tricarboxylic acid cycle pathways (Figure 2). Among them, six metabolites, including sedoheptulose-7-phosphate, fructose-1,6-diphosphate, phosphoenolpyruvate, α-ketoglutarate, succinate, and fumarate, were continuously up-regulated under MeJA elicitation, whereas the contents of seven other metabolites were generally down-regulated. In addition, the amino acid detection results indicated significant differences in the content of 18 out of 19 identified amino acids. Among them, 13 differential amino acids were generally down-regulated, while the remaining 5 differential amino acids, namely phenylalanine, tyrosine, tryptophan, proline, and alanine, showed a continuous upward trend (Figure 2 and Figure S2).

The continuous up-regulation of compounds such as sedoheptulose-7-phosphate, fructose-1,6-diphosphate, and phosphoenolpyruvate promoted the accumulation of aromatic amino acids, especially for phenylalanine, which is a crucial precursor for CGA biosynthesis. Interestingly, most of the primary metabolites showed a downward trend in this study. This may be due to the inhibitory effect of MeJA on primary metabolism, consistent with the previous report of a significant decrease in plant cell or tissue biomass after MeJA treatment [28,29].

Therefore, MeJA treatment led to a sustained increase in the levels of CGAs and their key precursors in *C. tinctorius* cells. To investigate the underlying mechanism of this promoting effect, transcriptome sequencing was further used to analyze the transcriptional response in the cells.

### 2.2. Overall Transcriptional Alteration of C. tinctorius Cells in Response to MeJA Treatment

#### 2.2.1. Results of Gene Annotation and New Gene Identification

Considering that transcriptional response precedes metabolite accumulation, this study analyzed the transcriptome of *C. tinctorius* cells in the early stage (6th h) of MeJA elicitation [including the MeJA-treated group (designated as MJ) and the control group (designated as CK)].

**Figure 3:**
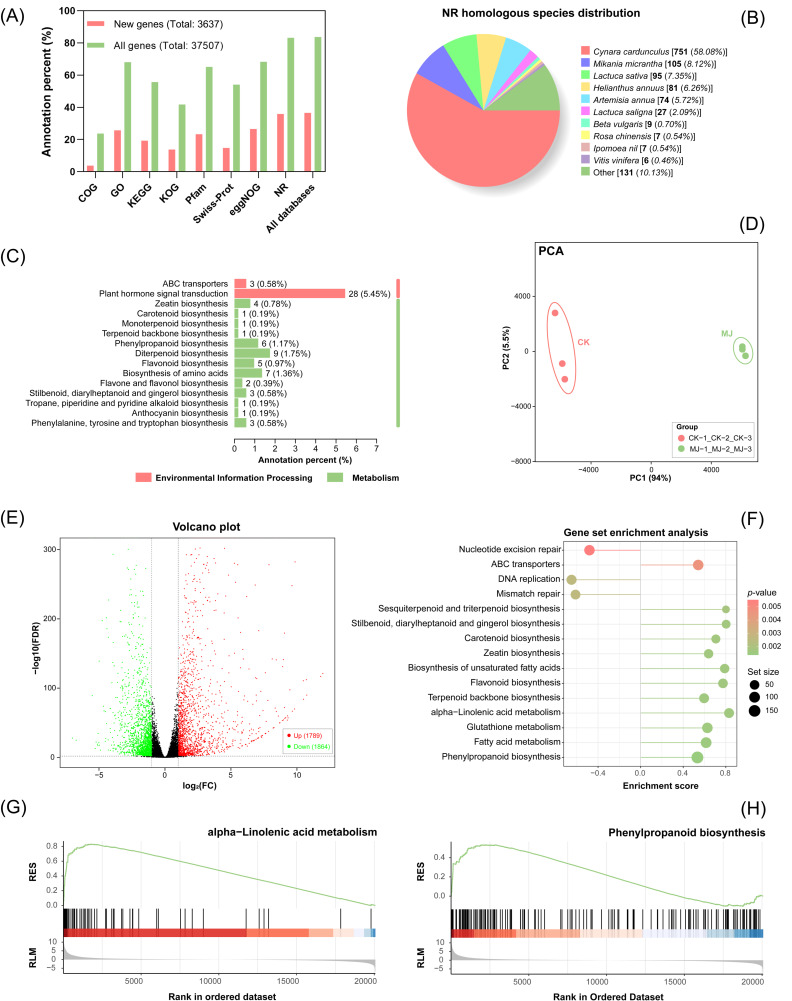
Overall transcriptional alteration in *C. tinctorius* cells exposed to MeJA: (**A**) summary of gene annotation; (**B**) homologous species distribution of new genes; (**C**) KEGG annotation on new genes; (**D**) principal component analysis of MJ and CK groups; (**E**) statistics of differential expression genes (black dots represent genes with non-significant differences); (**F**) top 10 enriched pathways in gene set enrichment analysis (GSEA); GSEA on (**G**) alpha-linolenic acid metabolism; and (**H**) phenylpropanoid biosynthesis pathways (RSE and RLM represent running enrichment score and ranked list metric, respectively).

The clean read numbers of the six samples ranged from 23,190,564 to 28,549,311, with genome annotation rates exceeding 90.86% for all samples (Table S1). The gene annotation results revealed a total of 37,507 genes, including 3637 new genes that were previously unannotated in the *C. tinctorius* genome (Figure 3A). Regarding the homologous species distribution of these new genes (Figure 3B), the highest sequence similarity was found in *Cynara cardunculus*, followed by *Mikania micrantha* and *Lactuca sativa*. In addition, a total of 75 new genes were categorized into pathways related to secondary metabolism (Figure 3C and Figure S3), with the plant hormone signal transduction pathway having the highest gene number. Notably, nine new genes were classified into pathways directly related to CGA biosynthesis, including phenylpropanoid biosynthesis and phenylalanine, tyrosine, and tryptophan biosynthesis pathways.

#### 2.2.2. Gene Differential Expression and Gene Set Enrichment Analysis

The clustering plot in the principal component analysis showed an obvious separation of the experimental group (MJ) and the control group (CK) based on their gene expression profiles, indicating a significant difference between MJ and CK (Figure 3D). The results of the gene differential analysis revealed 3653 differentially expressed genes (DEGs), comprising 1789 up-regulated genes and 1864 down-regulated genes (Figure 3E).

Gene set enrichment analysis (GSEA) enables enrichment analysis based on the expression of all genes. In contrast to conventional enrichment methods that focus only on significantly up- or down-regulated genes, GSEA also considers genes with low expression but consistent trends to identify biologically significant genes and pathways [30]. As shown in Figure 3F, the phenylpropanoid biosynthesis pathway was found to be the most significantly enriched pathway related to secondary metabolism in GSEA analysis. Additionally, the alpha-linolenic acid metabolism pathway was also significantly enriched. The running enrichment scores (RSE) of these two pathways were both greater than 0 (Figure 3G,H), and their peak RSE values were observed on the left side of the background gene sets. This indicated an overall up-regulation trend in the two pathways.

Thus, the transcriptional profiles of the alpha-linolenic acid metabolism and phenylpropanoid biosynthesis pathways, which are involved in jasmonate signal response and CGA biosynthesis, respectively, were significantly affected by MeJA elicitation. As explained in the following sections, further specific investigations were conducted on the above pathways and their extended pathways.

### 2.3. Transcriptional Differential Analysis of Endogenous Jasmonate Biosynthesis and Its Signaling Pathways in C. tinctorius Cells under MeJA Elicitation

**Figure 4:**
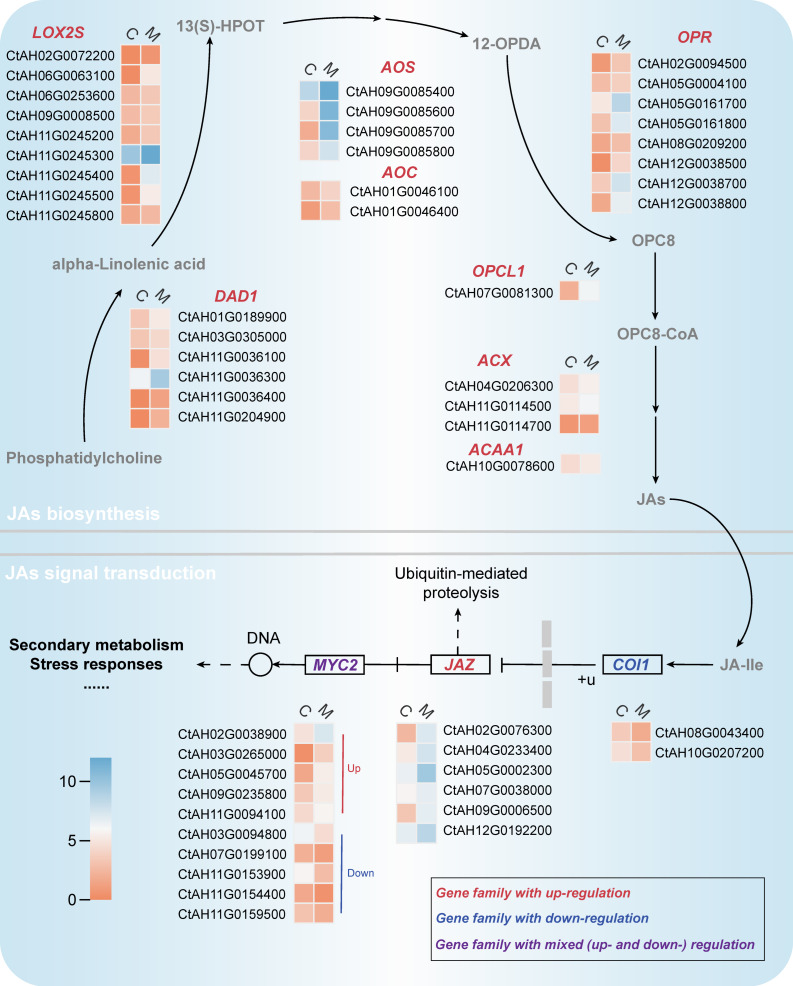
Transcriptional differential changes in jasmonate biosynthesis and its signaling pathways [C and M represent the *C. tinctorius* cells of the control group and MeJA treatment group, respectively. The rectangles represent the average FPKM of DEGs in different groups, which is normalized through the log_2_(FPKM value + 1)].

Endogenous jasmonates, which are key signaling molecules in response to environmental stress, originate from the alpha-linolenic acid metabolism pathway (Figure 4) [26]. In the endogenous jasmonate biosynthesis pathways, a total of 34 DEGs belonging to 8 gene families were identified, and all of them exhibited significantly up-regulated expression. Among them, the largest number of DEGs were categorized into the *13-lipoxygenase* (*LOX2S*) family (eight DEGs), followed by the *12-oxophytodienoate reductase* (*OPR*, eight DEGs) and *phospholipase A1* (*DAD1*, six DEGs) families.

*Coronatine-insensitive protein 1* (*COI-1*), *jasmonate ZIM domain-containing protein* (*JAZ*), and *Basic helix-loop-helix MYC2* (*MYC2*) are crucial elements of the jasmonate signaling pathway (Figure 4). Previous studies have shown that *MYC2* and *JAZ* interacted with the promoter regions of various transcription factors or structural genes, thereby regulating the secondary metabolism processes [31]. This study revealed 18 DEGs in the jasmonate signaling pathway. Among them, the expression of five DEGs in the *MYC2* family and six DEGs in the *JAZ* family showed up-regulation, while the expression of the other seven DEGs was down-regulated.

Therefore, MeJA significantly activated the jasmonate biosynthesis and its signaling pathways in *C. tinctorius* cells.

### 2.4. Transcriptional Differential Analysis of CGA biosynthesis Pathway in MeJA-Induced C. tinctorius Cells

#### 2.4.1. Transcriptional Changes in Shikimate Pathway

**Figure 5:**
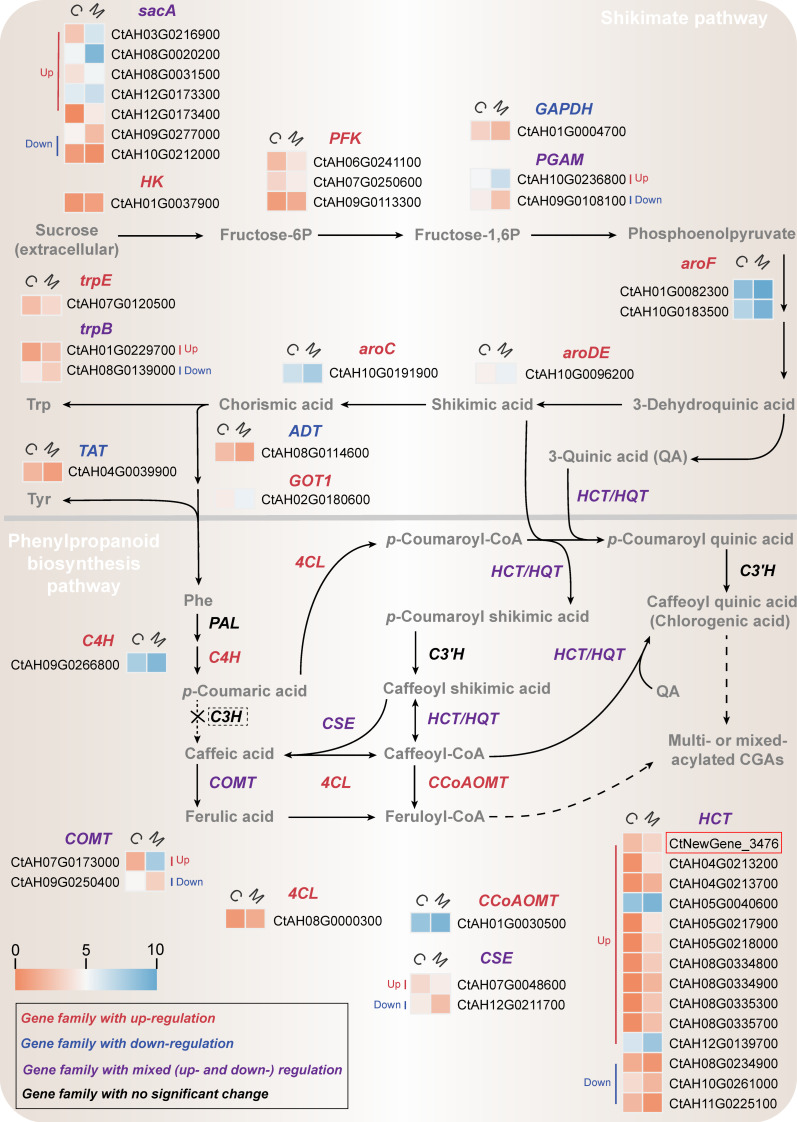
Transcriptional differential changes in the CGA biosynthesis pathway (the “X” symbol and dashed box are used to denote pathways that are hypothetically mediated by *C3H* and may not exist within the cell; the dashed arrows are used to indicate hypothetical pathways that are not yet fully confirmed).

CGAs are a class of branch products of the shikimate pathway and phenylpropanoid biosynthesis pathway (Figure 5). The former provides various key precursors for CGA biosynthesis, including phenylalanine, shikimic acid, and quinic acid. These precursors are further converted into CGAs through the phenylpropanoid biosynthesis pathway [15].

The results presented in Figure 5 demonstrate the identification of 24 DEGs (up: 19, down: 5) in the shikimate pathway, which encompasses 13 gene families. Among them, the *sucrose alpha-glucohydrolase* (*sacA*, up: 6, down: 1) family showed the most significant enrichment, followed by *6-phosphofructokinase 1* family (*PFK*, up: 2, down: 1). Moreover, significant up-regulation was observed in structural genes such as *3-deoxy-7-phosphoheptulonate synthase* (*aroF*, up: 2, down: 0), *3-dehydroquinate dehydratase/shikimate dehydrogenase* (*aroDE*, up: 1, down: 0), and *aspartate aminotransferase* (GOT1, up: 1, down: 0), which were directly associated with the biosynthesis of precursors for CGAs.

Therefore, the overall significant up-regulation of DEGs in the shikimate pathway promoted the rapid flow of sucrose into central carbon metabolism, thereby stimulating the biosynthesis of fructose-1,6-diphosphate, phosphoenolpyruvate, and other precursors for CGAs (e.g., phenylalanine, shikimic acid, and quinic acid). These results were consistent with the changes in intracellular metabolites described in Section 2.1.

#### 2.4.2. Transcriptional Changes in Phenylpropanoid Biosynthesis Pathway

The transcriptome annotation of *C. tinctorius* cells revealed a total of 92 genes from 8 gene families associated with phenylpropanoid biosynthesis pathway, including the *phenylalanine ammonia-lyase* (*PAL*), *cinnamic acid 4-hydroxylase* (*C4H*), *4-coumarate-CoA ligase* (*4CL*), *hydroxycinnamoyl transferase* (*HCT*), *CYP98A* (*C3’H*), *caffeoylshikimate esterase* (*CSE*), *caffeic acid 3-O-methyltransferase* (*COMT*), and *caffeoyl-CoA O-methyltransferase* (*CCoAOMT*) families (Figure S4). The *HCT* family had the highest number of annotated genes (43 genes), including 3 new genes that were not previously found in the *C. tinctorius* genome. However, genes from the *p-coumarate 3-hydroxylase* (*C3H*), *hydroxycinnamoyl-CoA: quinate transferase* (*HQT*), and *UGT84A20* (*UGCT*) families were not annotated in the transcriptome data. Therefore, three pathways for CGA biosynthesis may coexist in *C. tinctorius* cells, namely pathways 1, 2, and 3 (Figure 5 and Figure S1). The main pathway for CGA biosynthesis may be determined by the relative abundance of different hydroxycinnamoyl-CoA in the intracellular metabolic pool [19].

Differential expression analysis of the eight annotated gene families (Figure 5) showed that the *PAL* and *C3’H* gene families were not significantly differentially expressed, whereas 21 genes (up: 16, down: 5) belonging to the other 6 families displayed significant differential expression, indicating an overall up-regulation of the phenylpropanoid biosynthesis pathway. Among them, the *HCT* gene family had the maximum number of DEGs (up: 11, down: 3), representing 66.67% of the DEGs in the pathway. These results suggested a potential key role for the *HCT* gene family in MeJA-mediated biosynthesis of CGAs. In particular, the expression (FPKM value) of a newly annotated *HCT* gene (*CtHCT*, CtNewGene_3476) in the MeJA-treated group was 2.92 times higher than that of the control group.

#### 2.4.3. Correlation Analysis between the Newly Annotated *CtHCT* Expression and CGA Accumulation in MeJA-Treated *C. tinctorius* Cells

**Figure 6:**
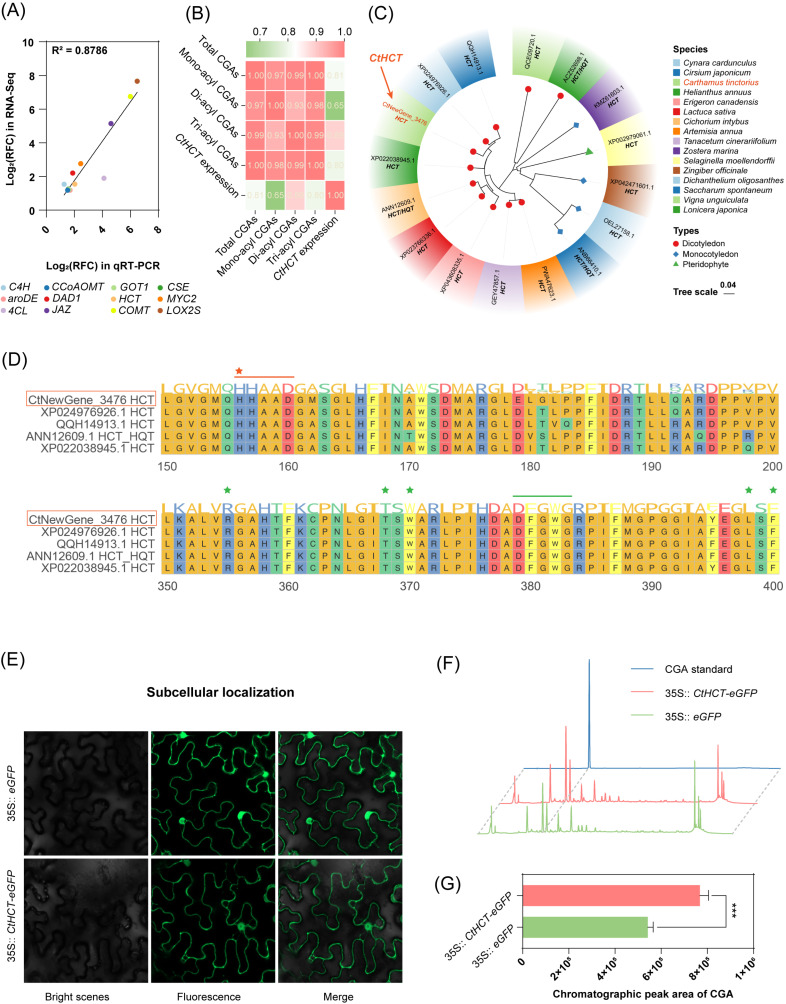
(**A**) qRT-PCR verification of transcriptional profile of DEGs in RNA-Seq (linear regression equation: Y = 1.169∗X − 0.5852; R and RFC represents correlation coefficient and relative fold change, respectively); (**B**) correlation analysis on RFC between the *CtHCT* (CtNewGene_3476) expression and CGA content in *C. tinctorius* cells induced by MeJA for different durations (24 h, 48 h, and 72 h); (**C**) phylogenetic tree analysis and (**D**) multiple sequence alignment of the *Ct*HCT protein (colored asterisks and lines represent the functional sites and conserved sequences of HCT proteins, respectively); (**E**) subcellular localization of the *CtHCT* gene, (**F**) HPLC analysis, and (**G**) chlorogenic acid (CGA) content comparison in *CtHCT*-overexpressed *N. benthamiana* (*** *p* < 0.001 indicates statistical significance levels from a *t*-test).

The qRT-PCR results revealed that the expression patterns of 12 key DEGs related to CGA biosynthesis, including the newly annotated *CtHCT* gene (CtNewGene_3476), were consistent with their transcriptional trends in the transcriptome data (linear correlation coefficient: 0.8786, Figure 6A and Table S2). In addition, the temporal expression trend of the *CtHCT* was positively correlated with the temporal changes in the total and subclasses CGA contents in MeJA-treated *C. tinctorius* cells, with Pearson correlation coefficients ranging from 0.6510 to 0.8870 (Figure 6B). 

These findings suggest that MeJA treatment significantly up-regulated genes related to CGA biosynthesis in *C. tinctorius* cells, resulting in a high level of CGA accumulation. Specifically, the MeJA-responsive *CtHCT* (CtNewGene_3476) might contribute significantly to this process. Therefore, further validations were performed to investigate the function of the *CtHCT* gene.

### 2.5. Analysis of Bioinformatics, Subcellular Localization, and Transient Expression of the Newly Annotated and MeJA-Responsive CtHCT

The transcriptome data showed that the transcript length of the *CtHCT* gene (CtNewGene_3476) was 2666 bp, consisting of two exons. Its open reading frame prediction revealed a protein-coding sequence of 1299 bp, encoding 432 amino acids (formula: C_2148_H_3323_N_563_O_622_S_19_; molecular weight: 47.60 kDa). Furthermore, the protein instability index, aliphatic index, and signal peptide count (Figure S5) were 36.33 (<40), 86.25 (<100), and 0, respectively, indicating its encoded protein is a stable, hydrophilic, non-secretory protein.

Phylogenetic analysis of the *Ct*HCT protein revealed a higher degree of sequence homology with HCT proteins from dicotyledonous plants in the *Asteraceae* family. However, it exhibited relatively lower sequence homology with HCT proteins from monocotyledonous and fern plants (Figure 6C). Multiple sequence alignment with the highly homologous sequences, such as QQH14913.1 (*Cirsium japonicum*), XP024976926.1 (*Cynara cardunculus*), XP022038945.1 (*Helianthus annuus*), and ANN12609.1 (*Cichorium intybus*), demonstrated that the *Ct*HCT protein contained the conserved domains of HXXXD (156–160) and DFGWG (379–383) for the HCT protein family (Figure 6D). The former domain is located in the catalytic active center of the HCT protein, while the latter is distal to the catalytic active center but still contributes to the catalytic process [32]. Therefore, the *CtHCT* (NewGene_3476) was a typical gene of the *HCT* family.

The subcellular localization analysis showed that the *CtHCT-eGFP* fusion gene produced significant green fluorescent signals in both the cytosol and nucleus of *N. benthamiana* (Figure 6E), in agreement with previous studies that have reported the cytoplasm as the main site of CGA biosynthesis [16]. Subsequently, transient overexpression of the *CtHCT-eGFP* fusion gene in *N. benthamiana* indicated a 41.56% increase in the relative CGAs (namely 5-caffeoyl quinic acid) content (Figure 6F,G). Therefore, the overexpression of the *CtHCT* (CtNewGene_3476) enhanced CGA biosynthesis in *N. benthamiana*.

### 2.6. In Vitro Catalytic Kinetic Analysis of the Recombinant CtHCT Protein

#### 2.6.1. In Vitro Catalytic Activity Assay

**Figure 7:**
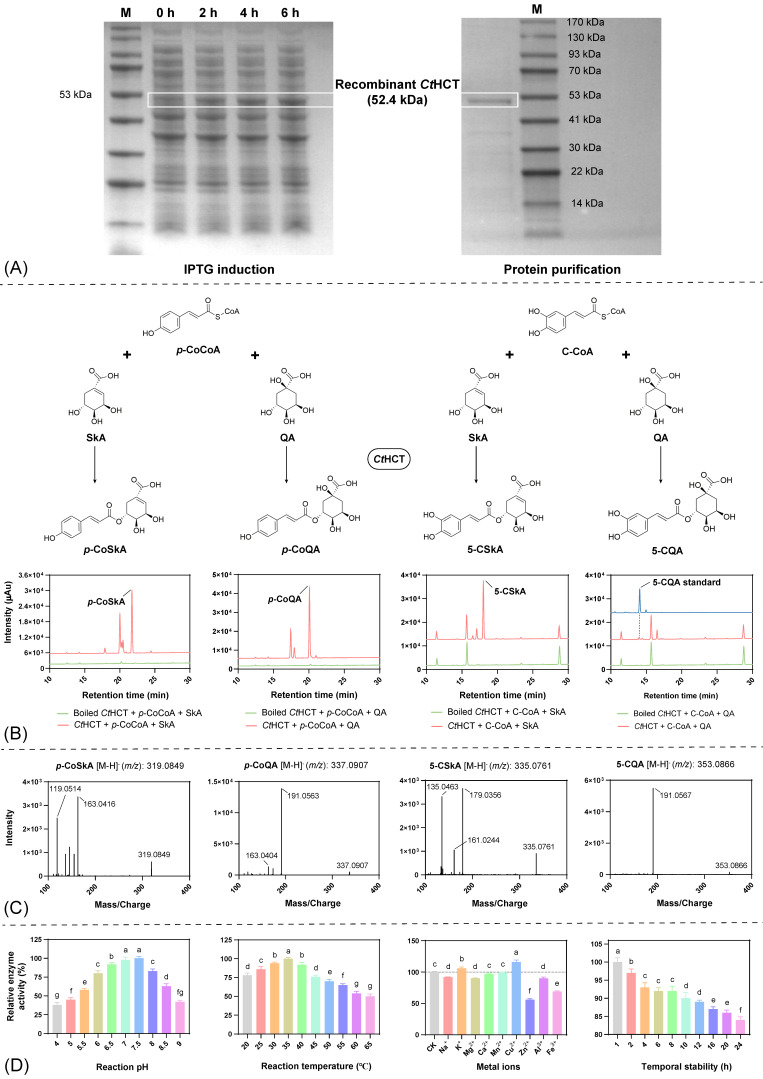
(**A**) Prokaryotic expression and purification of the recombinant *Ct*HCT protein (CtNewGene_3476); (**B**) HPLC and (**C**) UPLC-MS/MS detection of in vitro catalytic products of the recombinant protein (*p*-CoCOA, C-CoA, SkA, and QA represent *p*-coumaroyl-CoA, caffeoyl-CoA, shikimic acid, and quinic acid, respectively); (**D**) optimization of in vitro catalytic system conditions of the recombinant protein (Lowercase letters a–g represent the levels of significance as determined by Duncan’s new multiple range test, where different letters indicate a statistically significant difference (*p* < 0.05) between treatment groups).

The recombinant *Ct*HCT protein was expressed using the prokaryotic expression vector pET28a (+), which increased the theoretical protein size to 52.6 kDa due to the inclusion of His-tags and other functional sequences at both ends of the insertion site. As shown in Figure 7A, protein content in the target band region gradually increased with prolonged culture time after IPTG induction. Subsequently, Rosetta 2 (DE3) cells were induced with IPTG for 6 h and collected for protein purification, which removed most impurities and resulted in a single protein band near the 52.6 kDa region. Therefore, successful prokaryotic expression of the *Ct*HCT protein was achieved.

The in vitro catalytic activity of the recombinant *Ct*HCT protein was investigated in reaction systems with different acyl donors [*p*-coumaroyl-CoA (*p*-CoCOA) and caffeoyl-CoA (C-COA)] and acyl acceptors [shikimic acid (SkA) and quinic acid (QA)] (Figure 7B). HPLC analysis showed the appearance of new peaks in the chromatograms of all experimental groups when compared to the control group. Although the changes in the chromatograms were relatively less significant in the reaction system with C-COA and QA, the presence of new peaks was still observed. The reaction mixtures were analyzed by UPLC-MS/MS (Figure 7C), and precursor ion peaks with mass-to-charge ratios (*m*/*z*) of 319.0849, 337.0907, 335.0761, and 353.0866 were detected. By comparison with public literature data [24], they were identified as *p*-coumaroyl shikimic acid (*p*-CoSkA), *p*-coumaroyl quinic acid (*p*-CoQA), 5-caffeoyl shikimic acid (5-CSkA), and 5-caffeoyl quinic acid (5-CQA), respectively. Therefore, the recombinant *Ct*HCT protein exhibited catalytic activity for the formation of the corresponding hydroxycinnamic esters using different acyl donors (*p*-CoCOA or C-COA) and acceptors (SkA or QA).

#### 2.6.2. Reaction Condition Optimization

Optimizing reaction conditions for in vitro catalytic systems, such as reaction pH, temperature, presence of metal ions, and temporal stability, can enhance enzyme activity, reduce the use of expensive substrates, and lay the foundation for determining enzyme kinetic parameters. In terms of reaction pH and temperature (Figure 7D), the enzymatic activity of the recombinant *Ct*HCT protein exhibited an initial increase followed by a decrease with increasing values of these two parameters, with the optimal values being pH 7.5 and 35 °C. As for the presence of metal ions, Cu^2+^ and K^+^ significantly enhanced the enzymatic activity of the recombinant *Ct*HCT protein, with the maximum enzyme activity of 116% in the presence of 1 mM Cu^2+^. Conversely, the addition of Na^+^, Mg^2+^, Zn^2+^, Al^3+^, and Fe^3+^ ions resulted in a reduction in the enzyme activity to varying degrees. Among them, 1 mM Zn^2+^ had the most significant inhibitory effect, reducing the enzyme activity to 56%. In addition, the temporal stability of the recombinant *Ct*HCT protein showed that its activity gradually decreased over time, with 84% activity maintained at 24 h. Therefore, the optimized reaction conditions for the recombinant *Ct*HCT protein were as follows: reaction pH 7.5, reaction temperature 35 °C, addition of 1 mM Cu^2+^, and a reaction time within 1 h.

#### 2.6.3. Enzymatic Kinetic Analysis

Under the optimal reaction conditions, the enzymatic kinetic parameters of the recombinant *Ct*HCT protein were measured for different substrate combinations (Table 1). When QA was used as the saturating substrate, the Km value of *p*-CoCOA (9.41 µM) was lower than that of C-COA (32.61 µM), indicating that the recombinant *Ct*HCT protein had a higher affinity for *p*-CoCOA than for C-COA. Moreover, the *v*_max_/Km value of *p*-CoCOA (1.06 min^−1^) was much higher than that of C-COA (0.05 min^−1^), suggesting that the catalytic efficiency for *p*-CoCOA was 21.2 times higher than that for C-COA. Similarly, when *p*-CoCOA served as the saturating substrate, the Km value for QA (7.11 µM) was found to be lower than that for SkA (18.38 µM), and the *v*_max_/Km value of the recombinant *Ct*HCT protein for the former substrate (1.97 min^−1^) was 1.44 times higher than that for the latter substrate (1.37 min^−1^). Based on the enzymatic kinetic parameters, it was concluded that the *Ct*HCT protein preferred to use *p*-CoCOA and QA as substrates to produce *p*-coumaroyl quinic acid (*p*-CoQA), which was further converted to CGAs by other enzymes.

### 2.7. Revealing the Catalytic Mechanism of the CtHCT Protein by Molecular Docking

The homology modeling result demonstrated two similarly sized domains connected by a large crossover loop for the *Ct*HCT protein, with the catalytic center located in the cavity between the two domains (Figure S6). This spatial structure was similar to those of HCT proteins reported previously [32]. 

**Figure 8:**
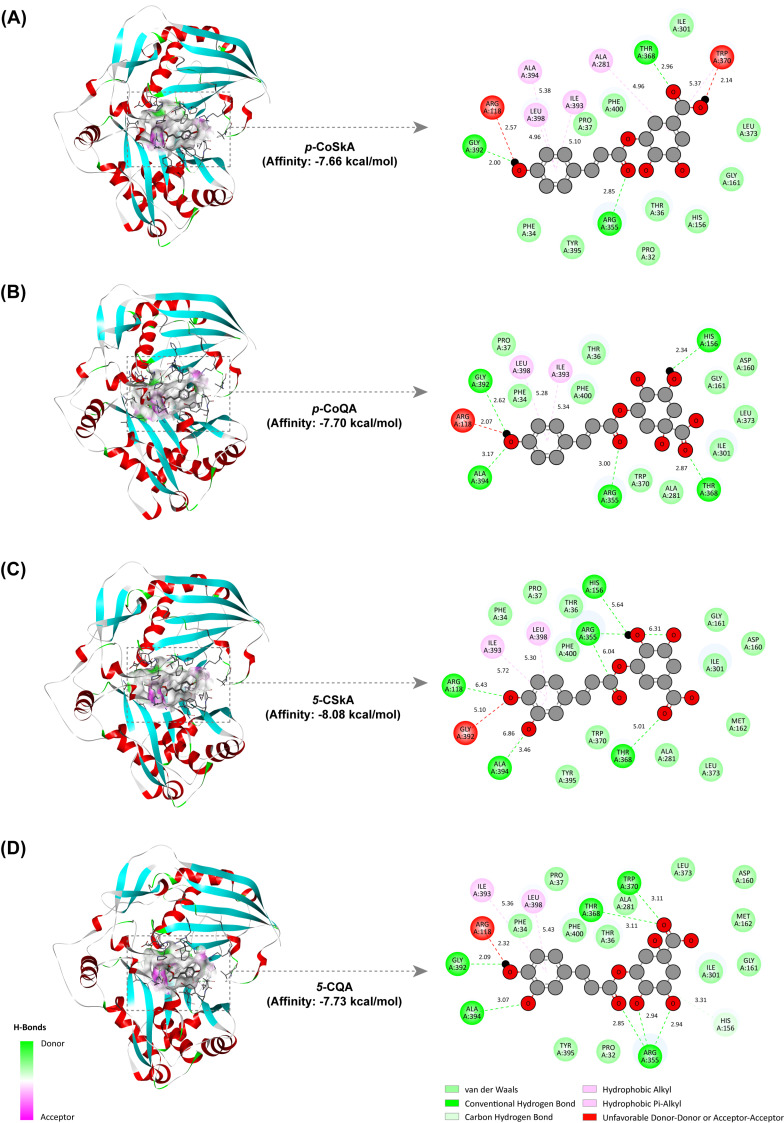
Molecular docking analysis between the *Ct*HCT protein (CtNewGene_3476) and different hydroxycinnamic esters [(**A**) *p*-coumaroyl shikimic acid (*p*-CoSkA); (**B**) *p*-coumaroyl quinic acid (*p*-CoQA); (**C**) 5-caffeoyl shikimic acid (5-CSkA); (**D**) 5-caffeoyl quinic acid (5-CQA)].

To elucidate the catalytic properties of the *Ct*HCT protein, a computational chemistry method was used to predict the interaction modes between the protein and the residues of the acyl donor (*p*-coumaroyl and caffeoyl residues) and acyl acceptor (shikimic acid and quinic acid residues) derived from different hydroxycinnamic esters. As shown in Figure 8, the four hydroxycinnamic esters identified in Section 2.6.1, including *p*-CoSkA, *p*-CoQA, 5-CSkA, and 5-CQA, all docked into the cavity between the two functional domains (domain I and II) of the *Ct*HCT protein, with binding energies ranging from −8.08 to −7.66 kcal/mol. This indicated that the binding between these hydroxycinnamic ester residues and the *Ct*HCT protein was spontaneous and stable. In terms of intermolecular forces, hydrogen bonds (conventional and carbon hydrogen bonds), van der Waals, hydrophobic forces (hydrophobic Alkyl and Pi-Alkyl), and repulsive forces (unfavorable Donor-Donor and Acceptor-Acceptor) were the main driving forces for the binding process. Specifically, these residues were capable of binding to the conserved site HIS156 of the protein catalytic center through hydrogen bonds or van der Waals. In addition, the amino acid residues in the *Ct*HCT protein, including ARG355, THR368, TRP370, LEU398, and PHE400, interacted with the residues of acyl donor and acceptor composing the hydroxycinnamic esters through hydrogen bonds, van der Waals, or hydrophobic forces (Figure 6D and Figure 8). 

Thus, these intermolecular interactions laid the foundation of spatial structure for the *Ct*HCT protein to catalyze the formation of various hydroxycinnamic esters, including CGAs.

## 3. Discussion

Our study identified previously unannotated genes involved in secondary metabolism in *C. tinctorius* cells. MeJA elicitation significantly up-regulated the expression and accumulation of genes and metabolites involved in the biosynthesis of CGAs in the cells. During this process, the newly annotated and MeJA-responsive *CtHCT* gene played a crucial role in stimulating CGA accumulation.

The *C. tinctorius* suspension cells in this study contained high levels of CGAs (approximately 10% DW), making the cells an excellent system for exploring functional genes involved in CGA biosynthesis. Previous studies have identified CGA biosynthetic genes in some medicinal plants and cultured cells. For example, de novo transcriptome assembly of a cDNA library in *Echinacea purpurea* yielded 85,736 unigenes, which were further used to identify CGA biosynthetic genes [15]. Similarly, the same strategy was utilized to investigate CGA biosynthetic genes in *Cecropia obtusifolia* and *Gardenia jasminoides* suspension cells, resulting in 118,756 and 57,069 unigenes, respectively [14,26]. Nevertheless, due to the absence of reference genomes, the de novo assembly strategy results in poor transcript completeness, an increase in duplicate transcripts, and the loss of low-expressed transcripts [33]. Recent advances in full-length transcript sequencing and genome assembly have led to the development of a chromosome-scale reference genome for *C. tinctorius*, enabling the accurate identification of functional genes in *C. tinctorius* [13]. However, differences in the physiological state and cultivation environment between the *C. tinctorius* plant and its suspension cells may generate unannotated and unique transcripts in the cells. The transcriptomic analysis of *C. tinctorius* cells identified 3637 new genes, 75 of which were involved in secondary metabolism pathways, particularly in CGA biosynthesis. These findings provided valuable information for the annotation of the *C. tinctorius* genome and paved the way for the investigation of its CGA biosynthesis.

Methyl jasmonate (MeJA) is a potent elicitor that regulates secondary metabolic processes, including CGA biosynthesis, by inducing complex and widespread transcriptional responses in plant cells [31]. During the investigation of MeJA-induced CGA accumulation in *Gardenia jasminoides* and *Echinacea purpurea*, exogenous MeJA was found to induce significant transcriptional changes in plant signaling, the biosynthesis of phenylpropanoid and its precursor, and transport-associated pathways [15,26]. However, the above reports did not address the effects of MeJA on downstream metabolites, particularly the changes in primary metabolites related to CGA biosynthesis. In this study, both transcriptional changes in CGA biosynthesis pathways and temporal changes in related primary metabolites were examined in MeJA-treated *C. tinctorius* cells, providing a comprehensive understanding of the regulatory role of MeJA in the high-level accumulation of CGAs in the cells. 

The *HCT* gene family has been reported to respond to MeJA elicitation. It was found that *CiHCT1* and *CiHCT2* from *Cichorium intybus* were directly related to the MeJA-induced accumulation of CGAs in its cell cultures [22]. To date, there have been a limited number of reports on the *HCT* gene family in *C. tinctorius*. For instance, transcriptomic analysis revealed that the *HCT* genes were significantly up-regulated in *C. tinctorius* inflorescences treated with MeJA, indicating their potential involvement in the process of MeJA-induced flavonoid accumulation in the inflorescences [11]. Furthermore, a series of *HCT* genes were identified in the *C. tinctorius* genome using the Hidden Markov Model, and their expression patterns were analyzed in MeJA-treated flowers at different developmental stages, revealing structural and expression differences among them [34]. However, the aforementioned studies were limited to transcriptomic or bioinformatic analyses and did not further validate the functions of the identified *HCT* genes in *C. tinctorius*. In this study, a positive correlation was observed between the expression of the newly annotated *HCT* gene (CtNewGene_3476) and the accumulation of CGAs in MeJA-treated *C. tinctorius* cells. Furthermore, the ability of this gene to mediate CGA biosynthesis was validated both in vivo and in vitro. Our findings provide insights into the role of the *CtHCT* gene in the biosynthesis of CGAs in the cells.

The recombinant protein of the *Ct*HCT (CtNewGene_3476) demonstrated the ability to catalyze the formation of various hydroxycinnamic esters, including CGAs, in vitro. However, due to the diverse enzymatic characteristics resulting from the substrate specificity and the preference of the *Ct*HCT protein, a comprehensive understanding of its catalytic mechanism is essential to unravel its role in the biosynthetic process of CGAs. In recent years, a series of studies have reported the composite crystal structures of HCT proteins with hydroxycinnamic ester products, such as *p*-coumaroyl shikimic acid. These studies have provided valuable insights into the catalytic mechanism of HCT proteins, refining our understanding of how these enzymes function [32,35]. Specifically, the initial step in the formation of *p*-coumaroyl shikimic acid involved the conserved histidine residue (His153) in the N-terminal domain of HCT protein, which interacted with the acyl acceptor (shikimic acid) through hydrogen bonding to facilitate deprotonation and initiate a nucleophilic attack with the acyl donor (*p*-coumaroyl-CoA). This resulted in the formation of a tetrahedral intermediate, which was stabilized by the threonine (Thr369) and tryptophan (Trp371) residues from the C-terminal domain through hydrogen bonding. Finally, protonation of the intermediate led to the release of coenzyme A and the formation of *p*-coumaroyl shikimic acid. Additionally, the residues of threonine (Thr369), arginine (Arg356), leucine (Leu393), and phenylalanine (Phe395) in HCT protein contributed to substrate recognition. Our docking analysis demonstrated that the *Ct*HCT protein interacted with various hydroxycinnamic esters through the residues corresponding to the above-mentioned active sites, indicating a similar catalytic mechanism for this protein. However, in vitro catalytic kinetics revealed that the *Ct*HCT protein had a lower ability to directly catalyze CGA generation and preferred producing *p*-coumaroyl quinic acid. This substrate preference may be attributed to the “arginine handle (Arg355)” and the surrounding amino acids [36]. With the assistance of enzymes such as C3’H, *p*-coumaroyl quinic acid and other hydroxycinnamic esters would be further converted into CGAs, which may explain the role of *Ct*HCT protein in mediating CGA biosynthesis in the cells.

This study provided the first elucidation of the regulatory mechanism of CGA biosynthesis in MeJA-treated *C. tinctorius* cells and the role of the MeJA-responsive *HCT* gene in boosting their CGA accumulation. These findings enhance our understanding of the regulatory mechanism of CGA biosynthesis, offering theoretical support for the efficient production of CGAs.

Although untranslated regions of gene sequences contain many functional elements for gene expression, the gene sequences obtained by chain-specific sequencing in this study lack these regions. Thus, full-length transcriptome sequencing can be used in the future to obtain the 5′- and 3′-end sequences of genes, enabling a more detailed investigation of the expression regulation of genes involved in CGA biosynthesis in *C. tinctorius* cells.

## 4. Materials and Methods

### 4.1. Product Induction of C. tinctorius Cells

Safflower (*C. tinctorius*) suspension cells were inoculated into B5 liquid medium at an inoculum of 10% (*m*/*v*) and cultured for 6 days at 28 °C and 115 rpm, as described in our previous study [8]. After filtration sterilization, methyl jasmonate [Sigma-Aldrich (Shanghai) Trading Co., Ltd., Shanghai, China] was added to the culture system at a final concentration of 100 µM and co-cultured for three days. The cells were harvested every 24 h for the subsequent detection of intracellular metabolites. B5 medium containing the following components was purchased from Qingdao Hope Bio-Technology Co., Ltd. (Qingdao, China): B5 basal salt (3.21 g·L^−1^), sucrose (30 g·L^−1^), α-napthaleneacetic acid (4 × 10^−3^ g·L^−1^), and 6-benzylaminopurine (2 × 10^−4^ g·L^−1^).

### 4.2. Intracellular Metabolites Detection

For the detection of CGAs in *C. tinctorius* cells, intracellular extracts were prepared and subjected to high-performance liquid chromatography (HPLC) analysis. The entire procedure was performed based on our previously reported methods [8] with some modifications in the elution procedure. The modified gradient elution procedure [(A) 0.05% (*v*/*v*) phosphoric acid solution and (B) acetonitrile] is as follows: 0–40 min, 5–41% B; 40–42 min, 41–90% B; 42–45 min, 90–5% B; 45–55 min, 5–5% B.

Intracellular primary metabolites, including carbohydrates, organic acids, and amino acids, were determined by gas chromatography-mass spectrometry. The quenching, extraction, derivatization, and detection of these primary metabolites were performed according to a previous study [27].

### 4.3. Transcriptome Sequencing and Analysis

#### 4.3.1. Transcriptome Sequencing

Samples from the cells treated with methyl jasmonate for 6 h (MJ) and the control group (CK) were selected for transcriptome sequencing, with three biological replicates in each group. Total RNA extraction, cDNA library construction, transcriptome sequencing (Illumina HiSeq platform, Ilumina, San Diego, CA, USA), and quality control of the above samples were performed with the technical support of Biomarker Technologies Co., Ltd. (Beijing, China) [37]. The raw data of transcriptome sequencing are available in the Sequence Read Archive database (https://www.ncbi.nlm.nih.gov/, accessed on 5 February 2024) with an accession number of PRJNA1072853.

#### 4.3.2. Gene Annotation and New Gene Identification

The alignment of clean reads from each library to the *C. tinctorius* genome (https://safflower.scuec.edu.cn/download.html, accessed on 1 May 2022) was conducted using HISAT2 software version 2.1.0 to obtain annotation information for known genes [38]. StringTie software version 2.2.1 was utilized to assemble mapped reads and compare them to the genome annotation information, resulting in the identification of previously unannotated transcript regions. The subsequent filtering of sequences with short peptide chains (number of amino acids < 50) or containing only a single exon enabled the discovery of new genes in *C. tinctorius* species [39]. The DIAMOND software version 2.0.15 was used to compare the newly annotated genes with sequences from main public databases to obtain their annotation information [40].

#### 4.3.3. Gene Expression and Differential Analysis 

FPKM (Fragments Per Kilobase of transcript per Million fragments mapped) was used as a metric to measure gene expression levels [37]. The |Fold Change| (≥2) and False Discovery Rate (<0.01) were set as key parameters in DESeq2 software version 1.10.1 to filter differentially expressed genes (DEGs) [41]. Additionally, clusterProfiler software version 4.10.0 was used to perform gene set enrichment analysis (GSEA) on KEGG pathways [42].

### 4.4. Gene Expression Patterns Validation

cDNA from the cell samples was obtained using MolPure^®^ TRIeasy™ Plus Total RNA Kit and Hifair^®^ AdvanceFast 1st Strand cDNA Synthesis Kit according to the manufacturer’s protocols (Yeasen Biotechnology Co., Ltd., Shanghai, China). Twelve differentially expressed genes in the RNA-seq data were subjected to qRT-PCR to validate their expression patterns, and a *ubiquitin C* gene was used as the internal reference gene. Their primers for qRT-PCR were designed using NCBI Primer-BLAST (https://www.ncbi.nlm.nih.gov/, accessed on 8 October 2023) and are listed in Table S2. qRT-PCR was performed with three replicates on a StepOnePlus™ Real-Time PCR System (ABI, Foster City, CA, USA) using a Hieff^®^ qPCR SYBR Green Master Mix Kit (Yeasen Biotechnology Co., Ltd., Shanghai, China). The relative expression levels of genes were calculated using the 2^−ΔΔCT^ method and then normalized.

### 4.5. Bioinformatics Analysis Methods 

The amino acid sequence of the newly annotated and MeJA-responsive *HCT* gene (*CtHCT*, CtNewGene_3476) was compared using NCBI Blastp to obtain information on sequence homology. The aligned sequences were analyzed using the Neighbor-Joining method in MEGA software version 7.0.26 to construct phylogenetic tree, which was then visualized using the online tool tvBOT [43]. Additionally, multiple sequence alignment analyses of the amino acid sequence of *Ct*HCT protein and its highly homologous sequences were performed using the ggmsa and ggplot2 packages in R studio software version 4.3.2.

### 4.6. Subcellular Localization and Transient Transformation

The cDNA of *C. tinctorius* cells was used as a template to amplify the coding sequence of the *CtHCT* gene (CtNewGene_3476) without a termination codon. The primer sequences are listed in Table S2. The *CtHCT* fragment was inserted between the Kpn I and Sal I sites of a modified binary vector pCAMBIA1300-*eGFP* to generate the fusion construct p1300-35S:: *CtHCT-eGFP*. The fusion construct was introduced into *Agrobacterium tumefaciens* GV3101 using the freeze-thaw method. Positive clones with kanamycin resistance were validated with PCR (Table S2) and used for transient transformation in *Nicotiana benthamiana* (*N. benthamiana*) following previously reported methods [16]. On day 3 after transformation, the fluorescence signals of *N. benthamiana* in the experimental (35S:: *CtHCT-eGFP*) and control groups (35S:: *eGFP*) were observed under a confocal microscope (LSM 980, Carl Zeiss AG, Jena, Germany). In addition, the CGA content of these groups was determined according to the methods described in Section 4.2.

### 4.7. Prokaryotic Expression and Purification of Protein

The *CtHCT* (CtNewGene_3476) encoding sequence without a termination codon was inserted between the BamH I and Not I restriction sites of the pET28a (+) plasmid, producing a fusion sequence with 6× His-tags at both the 3′ and 5′ ends. The construct was transformed into Rosetta 2 (DE3) competent cells (Weidi Biotechnology Co., Ltd., Shanghai, China), and positive clones were cultured in LB liquid medium containing chloramphenicol (34 µg·mL^−1^) and kanamycin (50 µg·mL^−1^) at 28 °C and 180 rpm until the OD_600_ value reached 0.6–0.8. Subsequently, isopropyl β-D-thiogalactoside (IPTG) was added into the cultures (final concentration: 0.5 mM) and co-cultured for 6 h. During this period, the expression of the recombinant protein was monitored by collecting cell samples at 2 h intervals and analyzing them using SDS-PAGE electrophoresis. 

Purification of the recombinant *Ct*HCT protein was performed using a His Label Protein Purification Kit (Beyotime Biotechnology Co., Ltd., Shanghai, China) as per the manufacturer’s instructions. After elution, the recombinant protein was exchanged into Tris-HCl buffer (100 mM, pH 7.5) using an ultrafiltration tube (Amicon^®^ Ultra-15 30K, Merck & Co., Inc., Rahway, NJ, USA), then mixed with an equal volume of glycerol, and stored at −80 °C. All reagents used in this section were purchased from Sangon Biotech Co., Ltd. (Shanghai, China).

### 4.8. In Vitro Enzyme Activity Assays and Product Identification

The volume of the in vitro catalytic reaction system is 200 µL, including 15 µg recombinant *Ct*HCT protein, 200 µM acyl donor (*p*-coumaroyl-CoA or caffeoyl-CoA), 400 µM acyl acceptor (shikimic acid or quinic acid), 1 mM 1,4-dithio-DL-threitol, and 100 mM Tris-HCl (pH 7.5). The reaction was carried out at 30 °C for 1 h, followed by the addition of 800 μL methanol to terminate the reaction. The reaction mixtures were centrifuged at 10,000× *g* and then used for the identification of their catalytic products with UPLC-MS/MS according to our previously described method [8]. In this part, *p*-coumaroyl-CoA and caffeoyl-CoA were purchased from Yuanye Bio-Technology Co., Ltd. (Shanghai, China), while other reagents were obtained from Solarbio Science & Technology Co., Ltd. (Beijing, China).

The relative catalytic activity (relative product content) of recombinant *Ct*HCT protein was analyzed using *p*-coumaroyl-CoA and shikimic acid as substrates. The analysis was performed under different conditions, including reaction pH (4–9), reaction temperature (20–65 °C), the presence of different metal ions (monovalent, divalent, and trivalent metal ions, 1 mM), and temporal stability (1–24 h). Under the optimal conditions, the catalytic kinetic parameters of the recombinant *Ct*HCT protein toward various substrates, including the Michaelis constant (Km) and the maximum reaction rate (*v*_max_), were calculated using Lineweaver–Burk plots [24].

### 4.9. Homologous Modeling and Molecular Docking

The amino acid sequence of the *Ct*HCT protein (CtNewGene_3476) was used to query the Swiss-Model web server, and the crystal model with the highest homology score (4g0b.1.A) was selected to predict a crystal structure of the *Ct*HCT protein [44]. Molecular docking between the *Ct*HCT protein and its substrates was performed using AutoDock Vina software version 1.1.2 [45], and the optimal docking conformations were visualized using DiscoveryStudio software version 2019.

## 5. Conclusions

Our findings highlighted *C. tinctorius* suspension cells as a promising platform for studying CGA biosynthesis regulatory mechanism. In addition, the newly annotated and MeJA-responsive *CtHCT* gene (CtNewGene_3476) was demonstrated to be involved in MeJA-induced CGA accumulation in the cells. Firstly, among the 3637 newly annotated genes in *C. tinctorius* cells, 75 were found to be associated with secondary metabolism. MeJA treatment significantly stimulated the expression of genes involved in plant signaling pathways and the biosynthesis of CGAs and their precursors, leading to the elevated accumulation of CGAs. Specifically, gene expression of the newly annotated and MeJA-responsive *CtHCT* was positively correlated with CGA accumulation in the cells. Furthermore, tobacco transient expression, in vitro catalysis kinetics, and molecular docking simulations confirmed that the *CtHCT* gene and its protein were capable of participating in the biosynthetic process of various hydroxycinnamic esters, including CGAs. Consequently, this study not only bridged the gap in annotation information within the *C. tinctorius* genome but also expanded our knowledge of the regulatory mechanism of CGA biosynthesis, establishing a theoretical foundation for high-yield CGAs in the cells.

## Figures and Tables

**Table 1:** Kinetic parameters of the recombinant *Ct*HCT protein.

Varying Substrate	Saturating Substrate	Km (µM) ^1^	*v*_max_ (µm/min) ^1^	*v*_max_/Km (min^−1^)
*p*-Coumaroyl-CoA	Quinic acid	9.41	10.02	1.06
Caffeoyl-CoA	Quinic acid	32.61	1.71	0.05
Shikimic acid	*p*-coumaroyl-CoA	18.38	25.12	1.37
Quinic acid	*p*-coumaroyl-CoA	7.11	13.98	1.97

^1^ Km and *v*_max_ represent Michaelis constant and maximum reaction rate, respectively.

## Data Availability

The raw data supporting the conclusions of this article will be made available by the authors upon request.

## Supplementary Materials

The supporting information can be downloaded at https://www.mdpi.com/article/10.3390/ijms25052710/s1.
