# Supplementary material for: Uncovering the Role of Hydroxycinnamoyl Transferase in Boosting Chlorogenic Acid Accumulation in Carthamus tinctorius Cells under Methyl Jasmonate Elicitation
Source: Int J Mol Sci. 2024 Feb 27;25(5):2710. doi: 10.3390/ijms25052710 (PMC10931740; doi:10.3390/ijms25052710)
Supplement: Supplementary file 1 [file ijms-25-02710-s001.zip › ijms-2886888-supplementary.pdf]

Supplementary Material

Table

Table S1 Statistical analysis of transcriptome sequencing data

Samples	Clean reads	Q30[1]	Mapped Reads
CK-1	23,190,564	92.95%	91.51%
CK-2	23,398,129	92.97%	92.37%
CK-3	28,549,311	92.42%	90.86%
MJ-1	25,432,291	93.56%	92.57%
MJ-2	25,783,350	92.95%	92.15%
MJ-3	25,268,504	93.70%	92.06%

[1] Q30 represents reads with a sequence accuracy greater than 99.99%; Mapped Reads represents reads annotated on the reference genome.

Table S2 Primers used in qRT-PCR and vectors construction

Primers	Primers' application	Primers sequences (5'→3')
DAD1-F	qRT-PCR	GACATCTCGAGGATCTGGCG
DAD1-R		TGAGCAAACTCGCCGTACTT
LOX2S-F		CGCCTACTCTCCGGTCATTC
LOX2S-R		CGGTTGGTTGGACGGTGATA
JAZ-F		GGAAACGCATCTTGGCAGTG
JAZ-R		TGTCCTCACGTTCGCAATGA
MYC2-F		GGAGTTCAACGAACAACGGC
MYC2-R		CGTCGAGATCTGAGTGGTCG
aroDE-F		TCATTGAGTAACGTGCCGCT
aroDE-R		GCACTGCCCCGTTTTCAAAT
GOT1-F		CCAAACGCCCCCTAAGTGAT
GOT1-R		ACGCCTACACGCCCATAAAA
C4H-F		AAAGGGCAGGACATGGTGTT
C4H-R		TGTTCGTGAAAAACGGCACC
4CL-F		ACCAACCCGGTGAGATATGC
4CL-R		CAGTGTGAAGCCAACCTTGC
HCT-F		GTTGACCCGAGAGCAACTCA
HCT-R		TGTACAGCTTGCTGTCCTGG
CSE-F		GCACATTCCACGTCAACCAC
CSE-R		CGTGATCGAGTTGCTCCTGA
CCoAOMT-F		GAAAACCGATCACATGGCGG
CCoAOMT-R		TAGCTCTTTCATGGGTGCCG
COMT-F		TGCAAATGCGTTGTCGATCC
COMT-R		ACCCTGTTGTGTGCAAGTGA
ubiquitin C-F		CAGCAACACCCCCTTTACTCT
ubiquitin C-R		AGGATGGAAATCGGGGTGGA
35S:: CtHCT-eGFP-F	Vector construction	ACGCGTCCCGGGGCGGTACCATGAAGATCGCCGTGACAGA
35S:: CtHCT-eGFP-R		AGCTCTGCAGGTCGACTTAGATATCGTACAAGAACTTGCT
pET28a (+)-CtHCT-F		AATGGGTCGCGGATCCATGAAGATCGCCGTGACAGA
pET28a (+)-CtHCT-R		TGCTCGAGTGCGGCCGCTTAGATATCGTACAAGAACTTGCT

Figures

Figure S1

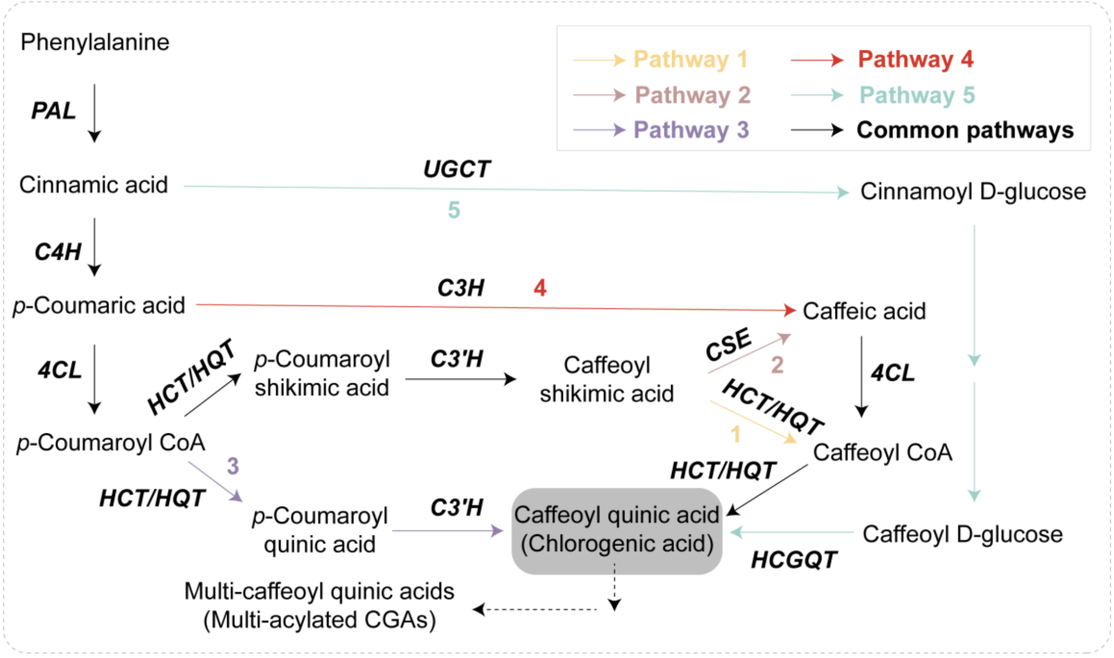

Figure S1 Biosynthetic pathways of chlorogenic acid in plants.

Figure S2

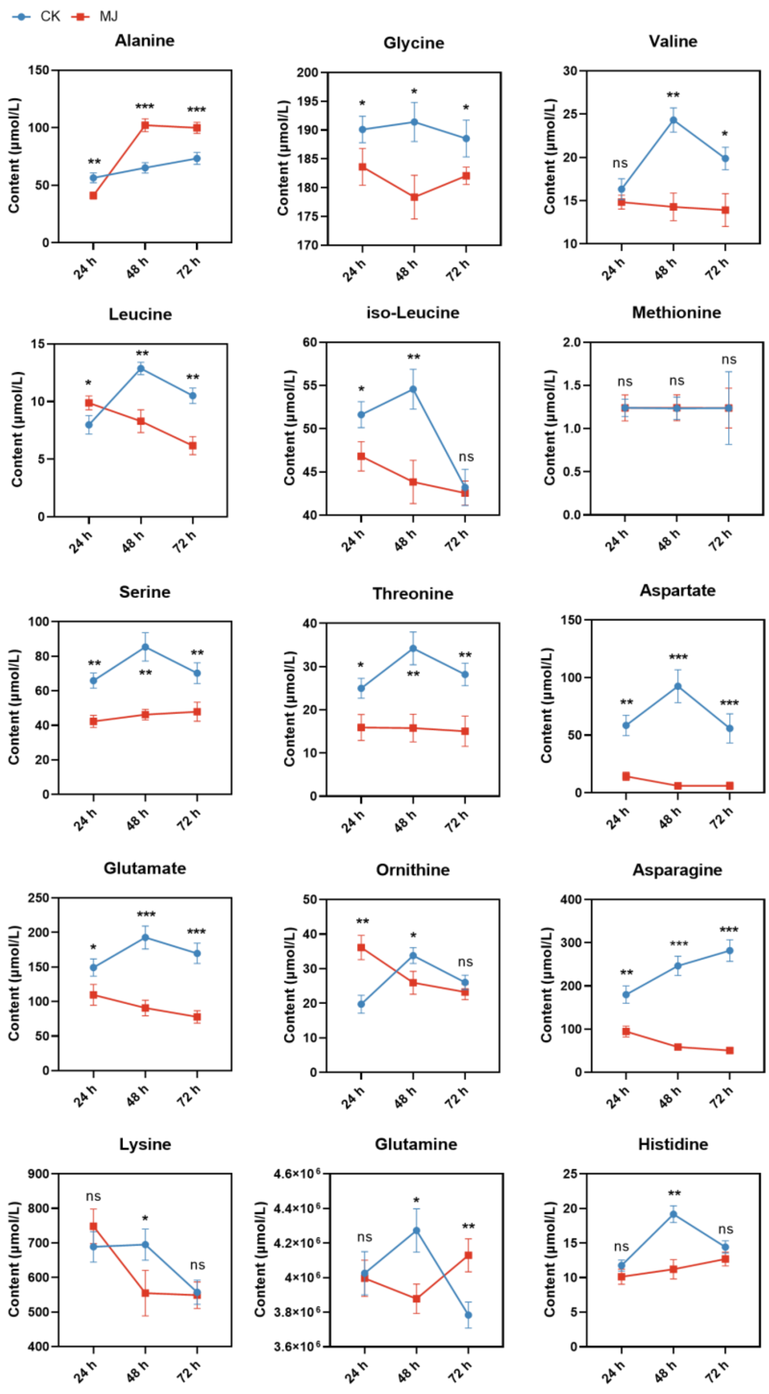

Figure S2 Temporal changes of amino acid in MJ-treated *C. tinctorius* cells.

Figure S3

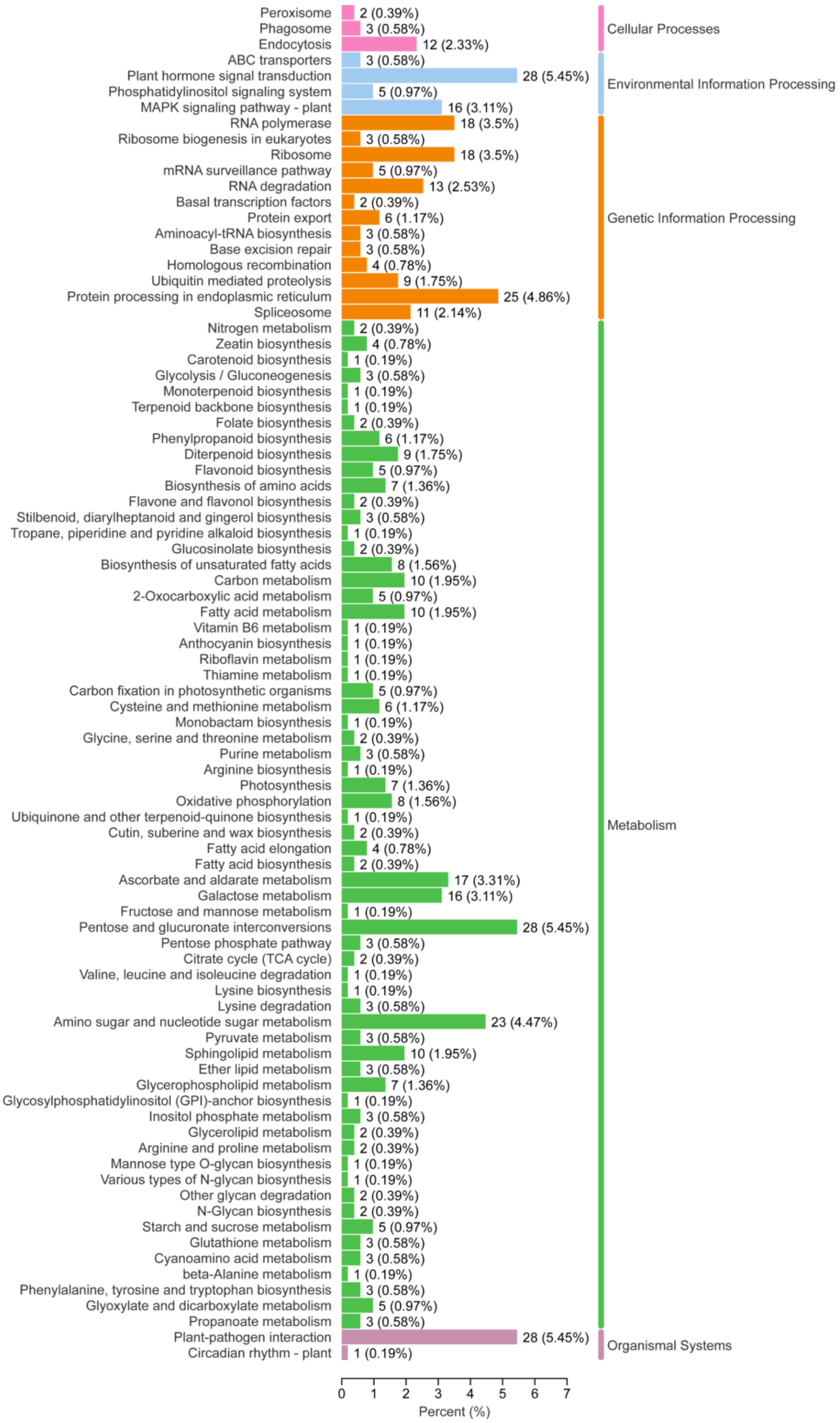

Figure S3 KEGG pathway annotation of new genes from *C. tinctorius* cells.

Figure S4

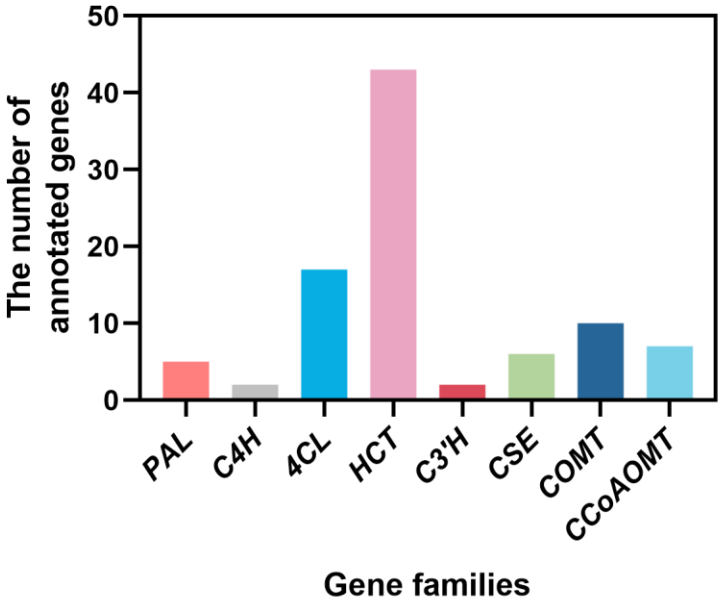

Figure S4 The annotation results of gene families involved in CGAs biosynthesis pathway in *C. tinctorius* cells.

Figure S5

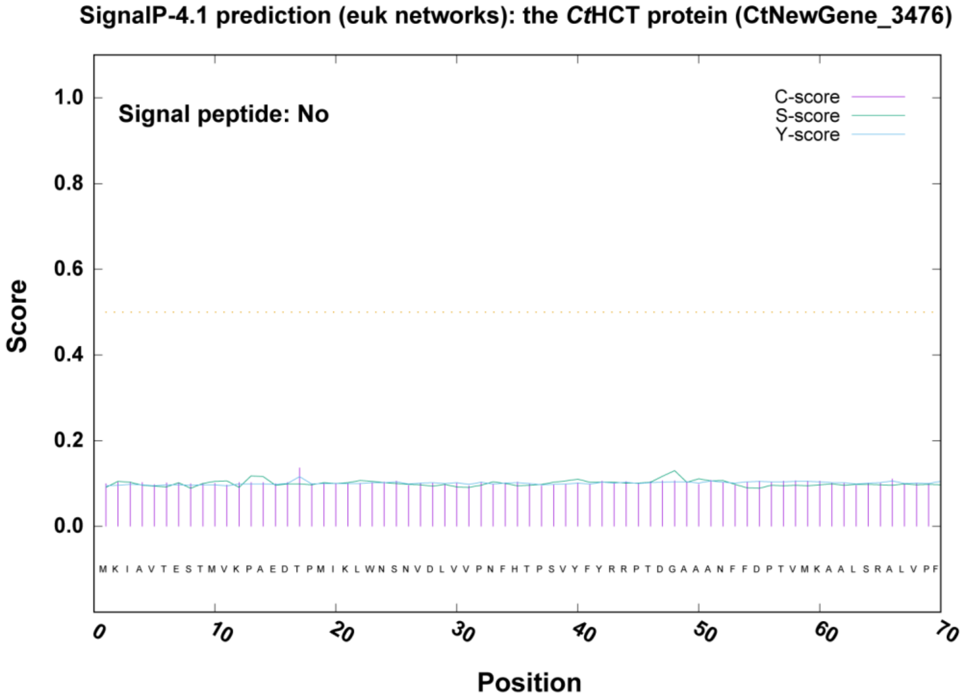

Figure S5 Signal peptide prediction (https://services.healthtech.dtu.dk/services/SignalP-4.1/) of the *Ct*HCT protein (CtNewGene 3476)

Figure S6

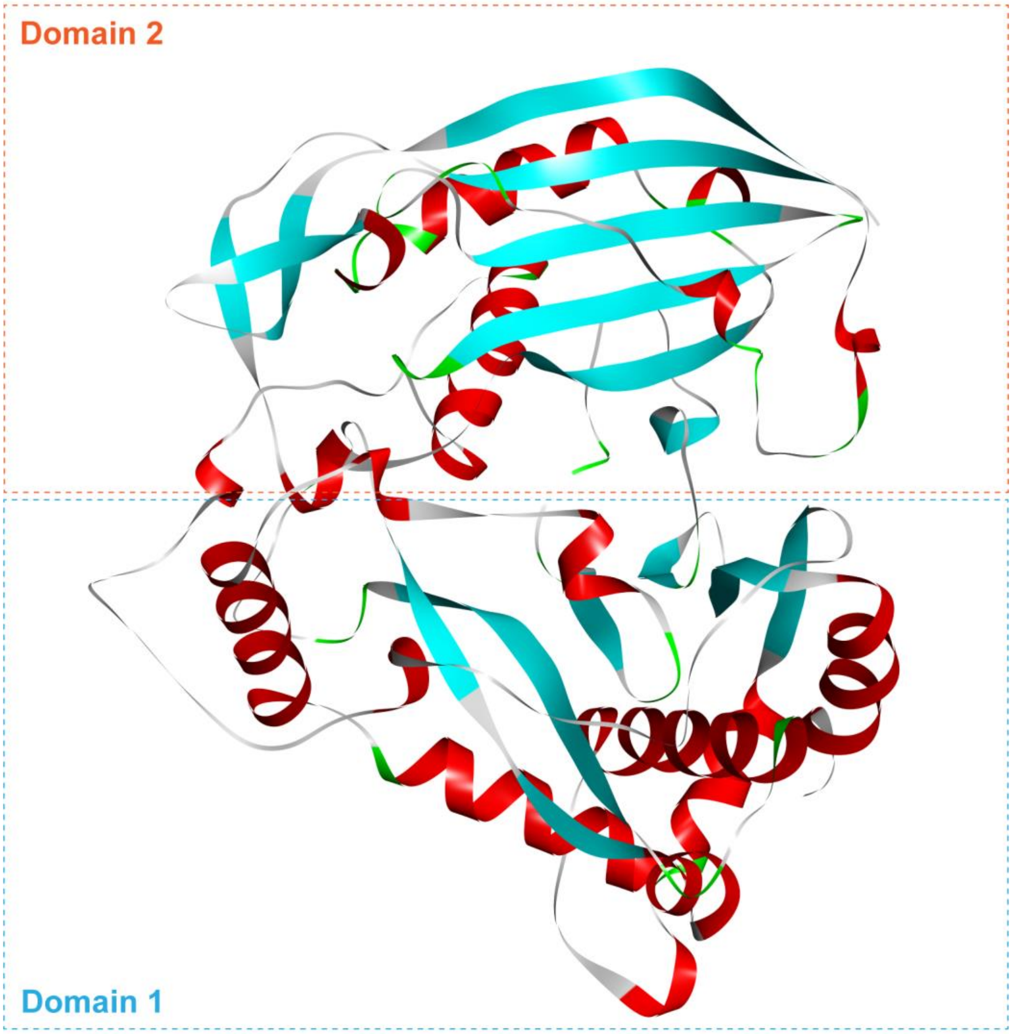

Figure S6 Protein structure prediction of the *Ct*HCT protein (CtNewGene 3476) through homologous modeling.
